# Better detection of platelet aggregation in patients with metabolic syndrome using epinephrine and ADP

**DOI:** 10.1186/1758-5996-6-93

**Published:** 2014-08-29

**Authors:** Laura Perez-Campos-Mayoral, Eduardo Pérez-Campos, Edgar Zenteno, Abraham Majluf-Cruz, Eduardo Perez-Ortega, Diana Matias-Pérez, Francisco J Rodal-Canales, Ruth Martínez-Cruz, Socorro Pina-Canseco, Miguel Angel Reyes Franco, Gabriel Mayoral Andrade, Pedro Hernández, Belem Gallegos

**Affiliations:** Centro de Investigacion UNAM-UABJO, Facultad de Medicina, Universidad Autonoma Benito Juarez de Oaxaca, CIMUU, Zaragoza, 213, Oaxaca, Mexico; Laboratorio de Patologia Clinica “Dr. Eduardo Pérez Ortega, Oaxaca, Mexico; Unidad de Bioquimica e Inmunologia Instituto Tecnologico de Oaxaca, Oaxaca, Mexico; Departamento de Bioquimica, Facultad de Medicina, UNAM, Kragujevac, DF Mexico; Unidad de Investigacion Medica en Trombosis, Hemostasia y Aterogenesis, IMSS, Mexico City, Mexico

**Keywords:** Platelets, Metabolic syndrome, ADP, Epinephrine

## Abstract

**Background:**

Patients with metabolic syndrome (MS) often have increased platelet aggregation. In order to determine which concentration detects a higher level of platelet aggregation in patients with MS, the agonists ADP and epinephrine were compared.

**Methods:**

The study included 56 subjects with MS and 53 healthy subjects. Blood pressure, weight, body-mass index, and hip-to-waist ratio were collected from all subjects. Insulin, glucose, total serum cholesterol, HDL-C, LDL-C, total triglycerides, markers of plasma atherogenicity, and indices of insulin resistance were measured in all participants. For aggregometry assays, the Born method was used. Platelets were treated with ADP and epinephrine in decreasing concentrations of 2.34, 1.17, and 0.58 μM, as well as, 11.0, 1.1, and 0.55 μM, respectively. ROC curves were plotted to define the diagnostic efficiency of epinephrine levels for MS.

**Results:**

Among healthy individuals and MS patients significant differences were observed in body weight, body-mass index, waist-circumference, levels of insulin, indices of insulin resistance, and levels of HDL-cholesterol, LDL-cholesterol and total triglycerides. There was a significant difference in the detection of increased platelet aggregation using 11.0 μM and 0.55 μM epinephrine and 0.58 μM ADP. With both agonists, ROC analysis showed an area under the curve of >0.8 for 11.0 μM epinephrine and 2.34 μM ADP. However, for MS patients, 11.0 μM epinephrine had a slightly better diagnostic efficiency than 2.34 μM ADP.

**Conclusions:**

It was found that 11.0 μM epinephrine and 2.34 μM ADP detected better platelet aggregation in patients with MS than in healthy subject. Both concentrations detected increased platelet aggregation in patients with MS.

## Background

Increased platelet aggregation is involved in myocardial infarction, ischemic stroke, type 1 diabetes mellitus, microangiopathy, and other diseases. For this reason, it is important to have effective methods for identifying this hyperactive state. Multiplate and VerifyNow are two other very widely used point-of-care- tests however, light transmission platelet aggregation (LTA), still remains the gold standard for the study of platelet function.

Platelet factors are involved, not only in the prothrombotic risk associated with insulin resistance [1], but also, in the risk associated with MS, which is related to some prothrombotic states of increased platelet aggregation, increased thrombin generation, and decreased fibrinolysis [2].

When comparing healthy individuals with patients of increased platelet aggregation, type 1 and type 2 diabetes mellitus, atherosclerosis [3], ischemic coronary disease, ischemic stroke [4], and several other vascular diseases is identified with the latter.

Diagnosis of increased platelet aggregation with LTA is performed by adding different concentrations of agonists to platelet rich plasma, such as: ADP, epinephrine [5], collagen, collagen-related peptide, arachidonic acid, or ristocetin [6]. In healthy subjects, Yee DL et al. reported that epinephrine and collagen-related peptides are reliable and efficient agonists for the detection of increased platelet aggregation [7].

The agonist epinephrine has a higher level of sensitivity and reproducibility for detecting increased platelet aggregation [3]; however, ADP is also used due to its ability to detect possible increments in P2Y12 activity [8]. Therefore, it is important to select the platelet agonist which is most appropriate for a particular clinical practice. In this study, the platelet agonists ADP and epinephrine were compared with the aim of identifying which concentration better detects increased platelet aggregation in patients with MS.

## Materials and methods

### Subjects and design

For this case–control study, patients with MS and healthy subjects between the ages of 25 and 75 were recruited over a period of three years. Sample size was calculated based on a 5% margin of error, a 95% confidence level, and a 50% response distribution (Raosoft, Herndon, VA) [9]. The case group had 56 MS patients, and the control group had 53 healthy subjects. Within these two groups, those who had taken antiplatelet or non-steroideal anti-inflammatory drugs during a 10-day period before entering the study were excluded.

The Joint Interim Statement (JIS) has a consensus criteria for the clinical diagnosis of metabolic syndrome [10], this is defined as a condition displaying three or more of the following indicators: greater waist-circumference according to ethnicity, ≥150 mg/dL triglycerides, < 40 mg/dL in males and < 50 mg/dL in females with HDL-C, ≥130 mm Hg systolic and/or ≥ 85 mm Hg diastolic blood pressure, and >100 mg/dL fasting glucose 4 [4]. Although the JIS does not consider insulin resistance within the criteria for MS, it recognizes that insulin resistance is a connecting factor in MS. The definition of insulin resistance is based on the homeostasis model assessment (HOMA-IR) [11]. In this study, all MS patients had at least three criteria within the JIS. In particular, waist-circumference was considered according to the following cutoffs: 94 cm for men and 90 cm for women [12].

Both MS patients and those in the healthy group were screened for insulin levels, assessed by the (HOMA-IR) [13], the quantitative insulin sensitivity check index (QUICKI) [14], and by the lipid-parameter-based index (Mffm/I) [15–17]. The study was approved by the Institutional Review Board of the Graduate Program and by the Ethics Committee of the Unidad de Bioquímica e Inmunologia del Instituto Tecnologico Regional de Oaxaca, México. Prior to entering the study, an agreement of written informed consent was obtained from all participants.

### Clinical and laboratory parameters

The following data was collected from all subjects: age, weight, height, hip and waist-circumferences, and body mass index (BMI). Height was measured with a standard scale and weight was measured using a digital scale.

Blood pressure (BP) was evaluated on three different occasions with a standard mercurial sphygmomanometer after lying in the supine position for 5 min. In addition, fasting glucose (Glc), total serum cholesterol (TC), HDL-C, low density lipoprotein cholesterol (LDL-C), and TT were measured with the Vitros DT60 II Chemistry System (Kodak, Rochester, NY, USA). Fasting insulin was tested with the Inmulite 1000 Immunoassay Analyzer (Diagnostic Products Corporation, Llanberis Glyn Rhonwy Caernarfon, UK).

### Platelet aggregation test

Blood was drawn from 53 healthy subjects and 56 patients with MS. First, it was diluted in a solution of 3.8% sodium citrate and centrifuged at 150 g, at room temperature for 15 min. Platelet-rich plasma (PRP) was removed with silicone-coated Pasteur pipettes and stored in plastic test-tubes at room temperature for a maximum of 180 min. Adenosine 5′-disphosphate (ADP) and epinephrine bitartrate were obtained from Sigma (Sigma Chemical Co., St. Louis, MO, USA). Various agonist concentrations were selected to identify possible differences within the groups. Concentrations of 2.34, 1.17, and 0.58 μM ADP and 11.0, 1.1, and 0.55 μM epinephrine were evaluated. LTA assays were performed on these agonist concentrations using a dual-channel platelet aggregometer (Sienco, Houston, TX, USA) based on the Born method [18]. Platelet counts of the PRP were standardized at 200 × 10^9^/L with autologous platelet-poor plasma. The incubation time was 1 min at 37°C. A stirring rate of 1,200 rpm and a running time of 6 min were maintained after adding 15 μL of the respective ADP and epinephrine concentrations.

### Calculations

The insulin sensitivity index, derived from fasting measurements of the HOMA-IR, QUICKI, and Mffm/I, gave an appropriate calculation of insulin resistance, which was calculated as previously described [6–8]. The Atherogenic index of plasma (AIP), a sensitive marker of differences in lipoprotein complex, increased significantly with greater atherogenic risk, this AIP was calculated as reported by Dobiásová [19].

### Statistical analyses

In consideration of the variability in factors occuring in subjects with insulin resistance, an attempt was made to select subjects with clinical characteristics as similar as possible. The Unpaired t test with Welch's correction was performed to detect differences in the median using the GraphPad Prism version 3.0 of Windows (GraphPad Software, San Diego, CA, USA). The p value < 0.05 was considered statistically significant, and the Receiver Operating Characteristic curve was plotted using the GraphPad Prism version 6.00 of Windows, GraphPad Software, La Jolla California USA, http://www.graphpad.com.

## Results

The groups evaluated in this study consisted of 53 healthy and 56 MS subjects. Amongst the two groups, significant differences were observed in terms of body weight, BMI, and waist-circumference. Additionally, significant differences were found in levels of HDL-C, LDL-C, TT, insulin, and AIP, HOMA IR, QUICKI, and Mffm/I indices (Table 1). This suggests that patients from the MS group are overweight, insulin resistant and have an increased atherogenic risk.

**Table 1:** **Demographic and biochemical characteristics of the study population**

	Healthy individuals	Patients with MS	P
	(n = 53)	(n = 56)	
Age (years)	63.0 ± 13.5	61 ± 16.0	0.4813
Weight (kg)	65.5 ± 13.0	80 ± 27.0	< 0.0001
SBP (mmHg)	121 ± 22.7	123 ± 40.0	0.7473
DBP (mmHg)	70 ± 7.9	70 ± 10.0	1.0000
BMI (kg/m^2^)	24.0 ± 1.98	27.0 ± 4.01	<0.0001
Waist-circumference (cm)			
Men	85 ± 4.2	105 ± 10.4	<0.0001
Women	75 ± 6.4	92 ± 5.7	<0.0001
Fasting glucose (mg/dL)	87.8 ± 7.79	88.8 ± 10.13	0.5635
AIP	0.38 ± 0.17	0.66 ± 0.26	< 0.0001
Insulin (μU/mL)	7.35 ± 3.08	9.78 ± 3.50	< 0.0002
HOMA-IR	1.57 ± 0.64	1.85 ± 0.78	0.0425
QUICKI	0.36 ± 0.02	1.13 ± 1.79	< 0.0022
Mffm/I	6.83 ± 1.18	5.11 ± 0.89	< 0.0001
TC (mg/dL)	232 ± 176	198 ± 62	0.1881
HDL-C (mg/dL)			
Men	47.5 ± 5.2	35.6 ± 16.2	<0.0001
Women	58.3 ± 6.5	36.1 ± 20.1	<0.0001
LDL-C (mg/dL)	106.40 ± 46.70	130.00 ± 60.60	0.0244
TT (mg/dL)	170.00 ± 45.00	286.00 ± 155.50	< 0.0001

*SBP* systolic blood pressure, *DBP* dyastolic blood pressure, *BMI* body mass index, *Hb* hemoglobin, *Ht* hematocrit, *AIP* atherogenic index of plasma, *HOMA-IR* homeostatic model assessment, *QUICKI* quantitative insulin sensitivity check index, *Mffm/I* lipid-parameter-based index, *TC* total serum cholesterol, *HDL-C* HDL- cholesterol, *LDL-C* LDL-cholesterol, *TT* total triglycerides, *MS* metabolic syndrome. All values were presented as mean ± SD. Differences between the groups were analysed using Unpaired t test with Welch's correction.

In relation to platelet aggregometry assays, an increase in platelet aggregation was found in healthy subjects with the addition of 1.17 μM ADP, and 1.1 μM Epinephrine. In MS patients, concentrations of 0.58 μM ADP, 11.0 and 0.55 μM epinephrine were increased (Table 2).

**Table 2:** **Results of platelet aggregometry assays**

	Healthy individuals	Patients with MS%	P
	(n = 53)	(n = 56)	
ADP 2.34 μM	61.4 ± 11.7	59.2 ± 8.9	0.2739
ADP 1.17 μM	53.8 ± 12.3	32.4 ± 19.9	0.0001*
ADP 0.58 μM	38.5 ± 8.85	43.5 ± 7.2	0.0017*
Epinephrine 11.0 μM	47.4 ± 15.0	84.0 ± 30.8	<0.0001*
Epinephrine 1.1 μM	36.4 ± 15.2	14.8 ± 17.3	<0.0001*
Epinephrine 0.55 μM	17.4 ± 4.7	26.8 ± 7.7	<0.0001*

*ADP* adenosine diphosphate, *MS* metabolic syndrome. All values are presented as mean ± SD. Differences between groups were estimated using a Unpaired t test with Welch's correction. *statistically significant.

**Figure 1:**
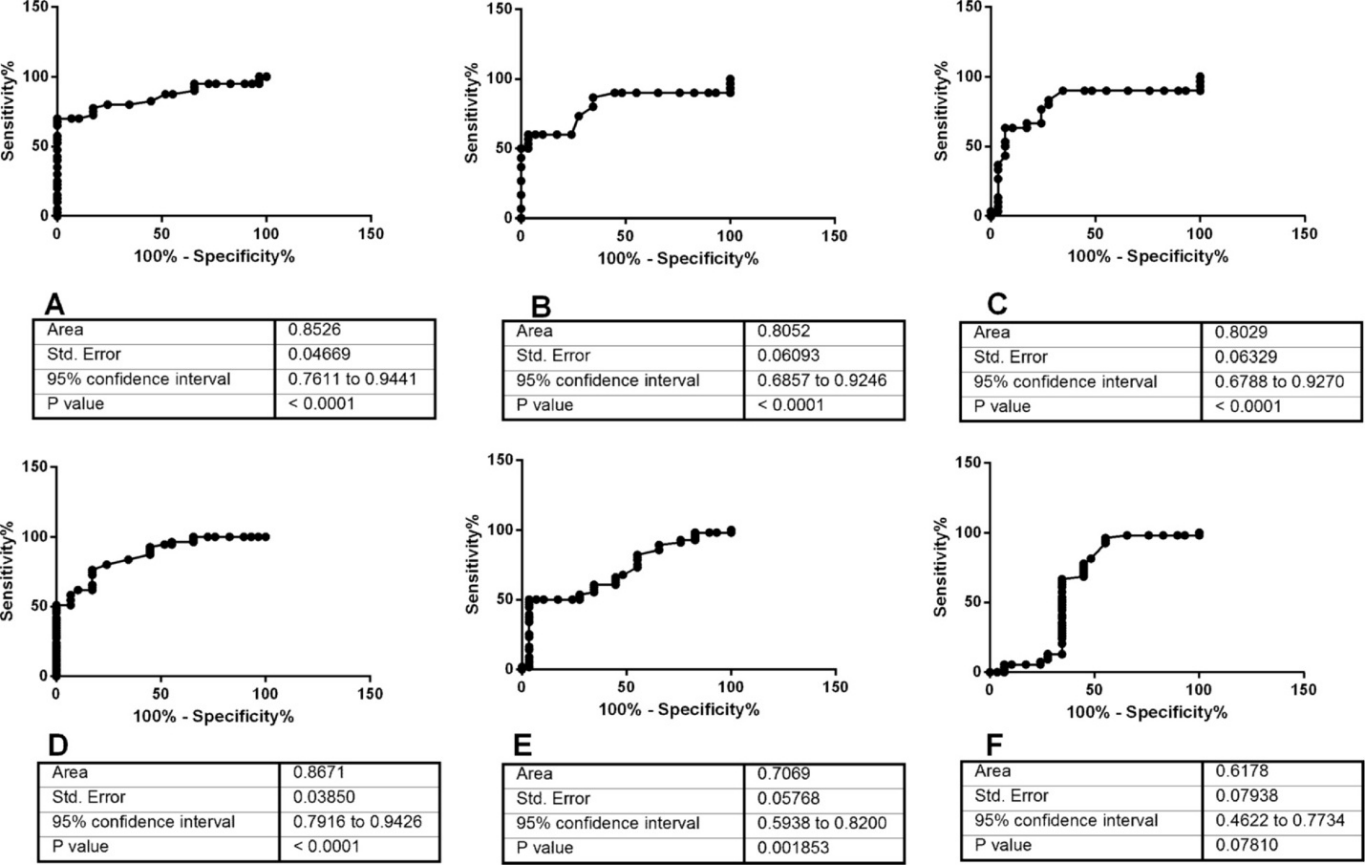
**Receiver operating characteristic curves derived from platelet aggregometry assays from healthy individuals and MS patients. A**. Epinephrine 11.0 μM. **B**. Epinephrine 1.10 μM. **C**. Epinephrine 0.55 μM; **D**. ADP 2.34 μM. **E**. ADP 1.17 μM. **F**. ADP 0.58 μM.

The ROC curve was used in order to differentiate between healthy subjects and patients with MS and to compare the effects of the concentrations of epinephrine and ADP. The area under the curve (AUC) is generally considered acceptable when >0.8 [20]. This study showed an AUC >0.8% for epinephrine and ADP concentrations of 11.0 and 2.34 μM, respectively (Figure 1).

Three different concentrations of agonists in ROC analysis of platelet aggregometry revealed that one, had a cut-off of > 57.0%, a sensitivity of 80.00%, and a specificity of 75.86% with 11.0 μM epinephrine, another, had a cut-off of <30.75%, a sensitivity of 60.00%, and a specificity of 89.66% with 1.1 μM epinephrine, and the other, had a cut-off of > 24.50%, a sensitivity of 33.33% and a specificity of 96.55% with 0.55 μM epinephrine. These results highlighted the differences between healthy and MS subjects.

ROC analysis of ADP revealed that a platelet aggregation cut-off of > 58.15% had a sensitivity of 76.36%, and a specificity of 82.76% with 2.34 μM. A cut-off of <30.75% had a sensitivity of 50.00%, and specificity of 89.00% with 1.17 μM, and an inverse cut-off of < 30.25% had a sensitivity of 5.55% and a specificity of 93.10% with 0.58 μM ADP. These analyses also showed differences between healthy and MS subjects.

## Discussion

Since increased platelet aggregation is related to atherothrombotic risk, it is important to identify subjects with persistent hyperfunctional platelets [21]. ADP is one of the most important platelet agonists. ADP is a nucleotide able to activate three different subtypes of receptors namely, P2Y1, P2Y12, and P2Y13 [22]. These receptors are also *in-vitro* concentration dependent. For example, ADP concentrations of between 10 and 20 μM may cause TxA_2_-independent aggregation while a moderate concentration of ADP (5 μM), only partially, depends on TxA_2_ [23]. Moreover, in order to initiate the agonist effect of ADP, co-activation of P2Y1 and P2Y12 is required. In this study, a low concentration of ADP was also used.

Noteworthy, is an increased response in platelet aggregation, elicited by ADP assays, due to a P2Y12 receptor gene H2 haplotype. This is also able to increase the risk of coronary artery disease [24]. Also, signaling, through the P2Y12 receptor, enhances platelet reactivity, due to hypercholesterolemia [25].

It appears that ADP and epinephrine are weak agonists in platelet aggregation, in contrast to thrombin and collagen, which are used as agonists in other methods to identify platelet hyperactivity associated with polymorphisms, such as, GPIIIa PlA1 allele, in the case of Collagen/Epinephrine closure time (CEPI-CT) [26].

Platelet hyperactivity in LTA with epinephrine is associated with ageing men [27], and also in patients with non-insulin-dependent diabetes mellitus (NIDDM). It is attributed to enhanced platelet aggregation in the presence of a thrombin receptor-activating peptide or epinephrine [28], and noted in studies in patients with ischemic heart disease, by Yokoyama M et al. [29]. The latter found that ADP and epinephrine had variable responses from person to person, using a range of concentrations from 100 to 0.1 μM, and an increased platelet sensitivity to adrenaline in subjects of advanced age. Epinephrine is also related to a variant of angina, which is detected by less agonist activity and relates to concentrations used in this study [30].

In this study, 11.0 μM epinephrine gave a ROC curve area of 0.8526, which suggests that this may differentiate better between healthy subjects and patients with MS. Moreover, 2.34 μM ADP gave a slightly larger area under a curve of 0.8671. Lesser doses of 0.55 μM ADP were not able to identify MS patients correctly, although shown as significant in Unpaired t tests with Welch's corrections (Table 2).

When comparing the ROC curves of 2.34 μM ADP and 11.0 μM epinephrine, although the area of the curve was greater in ADP, the epinephrine curve climbed quickly toward the top-left. This suggests that the 11.0 μM concentration may be better in identifying patients with increases in platelet aggregation. However, in consideration of the fact that both agonists provide information related to different signaling pathways, both concentrations may be useful in the study of MS.

Obesity and MS are strong predictors of coronary heart disease, in addition to the use of the AIP [31]. In the MS group there was an increase in the AIP. As a consequence, one may come to the conclusion that the group with MS also had an increased atherogenic risk and a higher risk of developing type 2 diabetes and vascular conditions when compared to the healthy group.

Moreover, individuals with high total triglycerides have greater AIP levels and an increased atherogenic risk. In the MS group, hypertriglyceridemia, with reduced HDL-C and increased LDL-C, is associated with the atherogenic triad, and may be related to increased platelet aggregation, leading to an increased atherothrombotic risk [32, 33].

In this study, 44 patients in the MS group were women. They had an waist-circumference increased, which suggests that, although not all patients with obesity have insulin resistance, they may have increased platelet aggregation as a consequence of the negative effects of visceral obesity [34]. Moreover, the anti-aggregating effect of insulin is lost on patients with insulin resistance as it can occur in obesity, diabetes mellitus, arterial hypertension [35], or in MS. In the MS group, despite having an increase in insulin, its effect on platelets was reduced.

The HOMA-IR, QUICKI, and Mffm/I indices are based on fasting levels of insulin and glucose. All indices in the MS group differed significantly, compared to those obtained in healthy subjects. These indices were an evaluation of hepatic insulin resistance (IR), rather than peripheral insulin sensitivity. Hepatic IR is a major contributing factor in pre-diabetic states and it is also related to impaired fasting glucose levels [36]. However, it is not totally proven if platelet insulin resistance is due, solely, to obesity [14, 37]. In this study, all the MS patients had an increased weight and obesity, which may indicate that these patients have platelet insulin resistance.

This study has some limitations and caution should be taken in the interpretation of the findings because of the small sample size of the study. Additionally, it may be possible that other platelet agonists, such as serotonin and submaximal doses of epinephrine induce platelet aggregation and they could be considered in further analysis [38].

This study supports the use of these agonist concentrations to perform platelet aggregation assays, as they could improve identification of increased platelet aggregation in patients with MS.

## Conclusion

We found an increase in platelet aggregation in subjects with MS when platelet aggregation assays were performed using 11.0 μM epinephrine and 2.34 μM ADP. These tests showed an area under the ROC curve which distinguishes between patients with MS and healthy subjects with increased platelet aggregation.

## Abbreviations

(BP): Blood pressure
(Glc): Fasting glucose
(TC): ,Total serum cholesterol
(HDL-C): HDL-cholesterol
(LDL-C): LDL-cholesterol
(TT): Total triglyceride levels
(ADP): Adenosine diphosphate
(AIP): Atherogenic index of plasma
(HOMA-IR): Homeostasis model assessment
(QUICKI): Quantitative insulin sensitivity check index
(Mffm/I): Lipid-parameter-based index
(ROC): Receiver operating characteristic.
